# Marine-Sulfated Polysaccharides Extracts Exhibit Contrasted Time-Dependent Immunomodulatory and Antiviral Properties on Porcine Monocytes and Alveolar Macrophages

**DOI:** 10.3390/ani12192576

**Published:** 2022-09-27

**Authors:** Caroline Hervet, Frédérick Bussy, Claude Le Goff, Déborah Ménard, Pi Nyvall Collén, Matthieu Le Goff, François Meurens, Nicolas Bertho

**Affiliations:** 1 BIOEPAR, INRAE, Oniris, 44300 Nantes, France; 2 OLMIX SA, 56580 Bréhan, France; 3 Department of Veterinary Microbiology and Immunology, Western College of Veterinary Medicine, University of Saskatchewan, Saskatoon, SK S7N 5E3, Canada

**Keywords:** algal extracts, pig/porcine/swine, PRRSV, trained immunity, antiviral, alveolar macrophage

## Abstract

**Simple Summary:**

Algal extracts have a real potential in terms of animal health, strengthening the interest in this natural resource. In pigs, respiratory complex syndrome significantly alters the wellbeing of the animals and threatens the economical sustainability of the sector. In the current study, we assessed various marine-sulfated polysaccharides (MSP^®^) extracts on two relevant cell populations in pigs, i.e., porcine monocytes and alveolar macrophages. Then, we analyzed the impact of the extracts on the infections of the cells by two important viruses. A modulation of the inflammatory response as well as some inhibitions of viral replication were observed. The type of effect observed was dependent on the extract, the experiment set-up, and the virus. The results obtained prompt us to further decipher the effects of algal extracts on the porcine health and open the door to future experiments, particularly in vivo experiments.

**Abstract:**

Porcine respiratory complex syndrome has a strong economic impact on the swine breeding sector, as well as a clear repercussion on the wellbeing of the animals, leading to overuse of antimicrobial molecules. Algal extracts used in short-term treatments are empirically recognized by farmers as having a positive effect on pigs’ health, however, their mechanisms of action are not well known and more research is needed. Herein we studied the short and median term impact of three algal extracts, in vitro, on the pro-inflammatory and antiviral responses of porcine primary blood monocytes and alveolar macrophages, as well as the susceptibility of the treated cells to infection by Porcine Respiratory and Reproductive Syndrome Virus (PRRSV) and the Aujeszky’s Disease Virus (ADV). All extracts presented a pro-inflammatory short-term effect, associated for two of them, with an inhibition of the PRRSV replication. Conversely, the three extracts presented an anti-inflammatory median term effect, with no impact on PRRSV replication. The observed immune modulation prompts us to test, in vivo, the anti-PRRSV action of algal extracts and strengthen the interest for this natural resource.

## 1. Introduction

Porcine respiratory viruses have a major impact on porcine health [1]. Amongst these viruses, some are frequently encountered in France such as Porcine Respiratory and Reproductive Syndrome Virus (PRRSV) while others such as Aujeszky’s Disease Virus (ADV or Suid Herpesvirus 1—SuHV-1) are now rare in porcine domestic populations although regularly present in other regions of the world. While some porcine viruses, such as Influenza Type A virus (IAV), are strictly affecting the respiratory tract other viruses such as PRRSV and ADV have a much wider tropism. ADV is a DNA virus from the *Herpesvirales* order. Similarly to most of the Herpesviruses, ADV establishes latent infection in the swine. It is endowed with multiple mechanisms to escape the immune response, among them ADV produces several molecules inhibiting type I interferon (IFN-I) productions and signaling (for review [2]). Conversely, ADV triggers a strong pro-inflammatory response through NLRP3 activation and IL1β and IL18 release. PRRSV is a single strand RNA virus from the *Nidovirales* order. It also arbores IFN-I inhibitory molecules [3], and the interaction of PRRSV with porcine macrophages triggered a fascinating evolutionary arms race in the use of long non-coding and micro miRNA [4,5]. Although not latent, PRRSV can infect animals for several weeks or months before the animal immune system eventually gets rid of the virus. Thus, pre-infection or early post-infection medicinal interventions that strengthen the immune system before the establishment of these two viruses might have a strong impact on swine herds health.

In addition to their direct effects on the porcine host, the viruses facilitate the development of secondary bacterial infections [1] which further complicate the situation and often require the use of antimicrobial interventions. With the increase in antimicrobial resistance in bacterial pathogens, antibiotic growth promoters were banned from the European Union in 2006 (IP/05/1687). National as well as international programs have been implemented to decrease the use of antibiotics and to find prophylactic and therapeutic alternatives [6].

Alternative prophylactic strategies harnessing the concept of nutraceutical functional food and feed [7] which enable an improvement of the innate immune response and the subsequent adaptive response are attractive and also definitely needed. Amongst prophylactic alternatives, some probiotics, plant extracts, bacteriophages and others have been assessed, some of them are already present on the market while others are still in the research phase. Marine algae are attractive alternatives given the fact they are the fastest growing plant organisms; they show a huge diversity and they have the capacity to produce a myriad of different compounds [8]. In some studies, assessing the nutritional properties of marine algae, it has been shown that green, brown and red seaweeds have interesting nutritional characteristics as well as antimicrobial and immunomodulatory properties [9,10,11,12]. Corino and collaborators have reviewed the biological functions of seaweeds and seaweed extracts in pig nutrition and more generally in pig health [13]. Various biologically active compounds have been identified in algae including sulfated polysaccharides named ulvan, fucan, and carrageenan. These compounds represent from 4 to 76% of the dry weight of the algae [14,15,16]. For the ulvan rich algal extracts obtained from the seaweed *Ulva lacinulata* (previously known as *U. armoricana*), immunoproperties have been clearly identified and some mechanisms of action described [11,17,18]. Ulvans are mostly composed of sulfated rhamnose residues linked to uronic acids [19]. It results in a repeated disaccharide unit, ß-D-glucuronosyl-(1,4)-ɑ-L-rhamnose 3-sulfate, named aldobiuronic acid [19].

The understanding of algal extracts action mechanisms remains in its infancy and more research is definitely needed. In chickens, *U. lacinulata* extracts activate directly heterophils and monocytes through TLR2 and TLR4 [20,21]. In a pig’s intestinal epithelial cell line, this same extract stimulates early cytokines expressions via TLR4 [18]. In addition to their potential for short-term immune activation, a possible impact of algal extracts on long term innate cells responses, also named immune training, could be anticipated considering the chemical structure of some extracts such as ulvans. Trained immunity [22] is still an emerging concept, involving a memory of the innate cells, which once trained will more efficiently oppose several related or unrelated infections. Immune training has been mainly studied for monocyte/macrophages, using fungal/bacterial inductors. After the first exposition to the inductor, trained cells will return to their resting basal state; however, they have acquired the capacity for a stronger response to a second related or unrelated challenge [22]. The response involves the release of higher amounts of pro-inflammatory cytokines. The opposite side of trained immunity is immune tolerance. In this case, after a first immunostimulatory treatment, a second related or unrelated challenge will trigger a lower immune response [23]. Epigenetic modifications are accountable for the memory of these functional adaptations. A first report recently described porcine monocytes training by BCG but not by *Saccharomyces cerevisiae* β-Glucans [24]. Thus, data about trained immunity in pigs are extremely scarce and evidence of trained immunity triggering by algal extracts are not available at this stage to the best of our knowledge. In the current article, we tested time-dependent immunomodulatory and antiviral properties of various marine-sulfated polysaccharides (MSP^®^) extracts on porcine monocytes and alveolar macrophages. The Ulva extract and its formulated product Searup^®^ are known to induce the activation of chicken heterophils and monocytes [20]. Searup^®^ and Ulva extracts differ regarding the concentration of Ulva extract and the presence of vitamins in Searup^®^. Less is known on the immune effects of the Solieria extracts, which are prepared using the red seaweed, *Solieria chordalis*.

Indeed, this subject is important since monocytes and alveolar macrophages (AM) are the main target of many respiratory viruses. The training of these immune cells with different algal extracts could be advantageous in order to better fight these viruses.

## 2. Materials and Methods

### 2.1. Monocytes

Approximately 50 mL of blood from 3- to 6-month-old animals were sampled in heparinized tubes during the normal course of husbandry in a conventional breeding herd (Large white/Landrace/Pietrain), presenting a good health status (Brittany, France). Peripheral blood mononuclear cells (PBMC) were isolated using Ficoll density gradient (Lymphocytes separation medium, Eurobio Scientific, Les Ulis, France). One mL per well of PBMC at 8 × 10^6^ cells/mL in Roswell Park Memorial Institute (RPMI) 1640 media with GlutaMAX supplement (RPMI, Thermofisher Scientific, Saint-Herblain, France), supplemented with penicillin, streptomycin and 10% fetal calf serum (FCS) (complete medium), were then incubated 1 h in a 6 well plate. Non-adherent cells were flushed, and monocyte-enriched adherent cells were then treated with MSP^®^. 

### 2.2. Alveolar Macrophages

Lungs were issued from Large white animals, aged between 6 and 7 months, euthanized in the course of the regular management process of the *Unité Expérimentale de Physiologie Animale de l’Orfrasière* (UEPAO, Tours, France) presenting a highly controlled health status. A broncho-alveolar lavage (BAL) procedure was then performed twice on the isolated left lung with 250 mL of phosphate-buffered saline (PBS) supplemented with 2 mM Ethylenediaminetetraacetic acid (EDTA) (PBS/EDTA). BAL were centrifuged and resuspended in complete medium. One mL per well of BAL cells at 8 × 10^6^ cells/mL were incubated for 2 h in 6 well plate and mixed by pipetting after 1 h incubation time. Non-adherent cells were then flushed, and BAL cells enriched in adherent AM were treated with MSP^®^.

### 2.3. Virus Productions

Porcine Reproductive and Respiratory Syndrome virus type 1 (PRRSV-1) strain PRRS-FR-2005-29-24-1 (*Finistère* strain; genotype 1.1) was propagated on AM cultured in RPMI 1640 medium supplemented with 10% FCS and 1% Streptomycin/Penicillin/Amphotericin (SPA) solution (Eurobio scientific, Les Ulis, France) for 72 h. The supernatant was clarified by centrifugation for 20 min at 600× *g* and then purified on Amicon Ultra-15 centrifugal Filters (Sigma-Aldrich, Saint-Quentin Fallavier, France) after a 20 min centrifugation at 4000× *g* and 4 °C. The PRRSV-1 titer using Tissue Culture Infectious Dose (TCID50) assay protocol on AM was 8.7 × 10^6^ TCID50/mL. The Aujeszky’s disease virus (ADV) strain Kojnok propagation was p45to5m4r on Newborn Pig Trachea (NPTr) cells [25] in DMEM medium supplemented with 10% FCS and 1% of SPA solution (Eurobio scientific). The ADV titer using TCID50 assay protocol on Madin-Darby Canine Kidney cell line (MDCK) was 3.7 × 10^7^ TCID50/mL.

### 2.4. Marine-Sulfated Polysaccharides

Green tide algae *Ulva* sp. were collected on the beach at Plestin les Grèves (Brittany, France) and the biomass of the bulk samples analyzed contained a mixture of two foliose *Ulva* species, predominantly *U. lacinulata* Kützing (previously known as *U. laetevirens* Areshoug or *U. armoricana* Dion, Reviers & Coat [26]), and a lower amount of *Ulva* sp. A (previously known as *U. rigida* [27]). Algae were molecularly characterized by Pristine coasts company.

The red seaweed *Solieria chordalis* were collected on the beach at Saint-Hilaire-de-Riez (Vendée, France). The algae were washed in fresh water, drained and wet ground. The liquid and solid phases were separated as part of an industrial process (Patent No. FR 61909) and further processed by filtration. The seaweed extract compositions (see Table 1) were examined in triplicate using the following methods. Ash values were determined gravimetrically after incineration of samples at 550 °C for 6 h. The percentage elemental analysis (C, N and S) was carried out using “Elementar Analysensysteme GmbH-vario ELIII Element Analyzer” (Hanau, Germany) according to the manufacturer recommendations. 

### 2.5. Treatments

Cells were treated with (i) Searup^®^ (Olmix SA, Bréhan, France), a commercial product composed of the *Ulva* fraction formulated with vitamins A (850,000 UI/kg) and D3 (200,000 UI/kg) according to the European legislation (EU 2015/724 and EU 2017/1492), (ii) the non-formulated *Ulva* fraction (Ulva extract), and (iii) the non-formulated *Solieria* fraction (Solieria extract).

### 2.6. Marine-Sulfated Polysaccharide Treatment of the Porcine Cells and Virus Infections

Preliminary experiments were carried out in order to determine the highest non-toxic concentrations than can be used on porcine monocytes and AM. A dilution of 1/1000 of the primary extracts in RPMI + PS was chosen according to the similar cell counts observed in control and MSP^®^ treated cells after 24 h treatment or 24 h treatments plus 6 days of culture in complete medium + SPA.

In vitro experimental set up of MSP^®^ short- and medium-term treatments were adapted from Byrne et al. and Ifrim et al. [24,28]. Briefly, after 24 h MSP^®^ treatment, adherent monocytes or AM were washed twice with 2 mL of RPMI. To determine the short-term effect of MSP^®^ treatment the cells were stimulated with 10 ng/mL lipopolysaccharide (LPS) or Polyinosinic:polycytidylic acid (Poly IC) (Sigma-Aldrich) in RPMI supplemented with penicillin and streptomycin (PS). Twenty-four hours later, media were removed, and cellular RNA were collected by lysing the adherent cells with 2 × 300 µL of RLT Buffer (Qiagen, Courtaboeuf, France). Alternatively, one well of 24 h-MSP^®^ treated cells was counted, and the viral infectious doses were adjusted according to preliminary experiments (data not shown) in order to obtain measurable virus replications. Monocytes were inoculated with an identical multiplicity of infection (MOI) of 0.1 for PRRSV and ADV. AM were infected at MOI = 0.001 for PRRSV and at MOI = 0.01 for ADV. Viruses were diluted in 200 µL RPMI and PS (complete RPMI), for 2 h, mixing every 15 min. The cells were then washed twice to remove the inoculum and cells were cultured in complete media + PS for 48 h. Media were then removed and cellular RNA were collected by lysing the adherent cells with 2 × 300 µL RLT Buffer.

To determine the medium-term effect of MSP^®^, after the 24 h MSP^®^ treatment and the washing, cells were cultured in complete RPMI for 7 days with complete media changes at 3 and 6 days. Then, monocytes or AM were stimulated with LPS or Poly IC as above for short-term cultures. Since cultured monocytes and AM are more susceptible to PRRSV and ADV infections than fresh cells (see results), 8-day-cultured cells were infected for only 24 h instead of 48 h for the short-term cultures, although using the same MOI (PRRSV Monocytes, MOI = 0.1 and AM, MOI = 0.001; ADV Monocytes MOI = 0.1 and AM MOI = 0.01). RNA was collected as above for short-term cultures.

### 2.7. RNA Extractions and Reverse Transcription (RT)-Quantitative Polymerase Chain Reaction (qPCR)

Total RNA in RLT Buffer was extracted using RNeasy MicroKit (Qiagen) following the manufacturer’s instructions. Total RNA quantity and quality were assessed using Nanophotometer (Implen, Munich, Germany). cDNA was generated with a reverse transcriptase in the iScript Reverse Transcription Supermix for RT-qPCR (Bio-Rad, Hercules, CA, USA) from 20 to 300 ng of RNA free of genomic DNA per reaction for monocytes and from 200 to 400 ng for AM.

The generated cDNAs were then diluted (2×) and combined with the primer set and SYBR Green Supermix (Bio-Rad) following the manufacturer’s recommendations. To carry out the qPCR assays the selected conditions were 98 °C for 30 seconds (s), followed by 40 cycles with denaturation at 95 °C for 5 s and annealing/elongation for 30 s at optimal temperature—depending on the chosen target (see Table 2).

The viral genomes were quantified by semi-quantitative RT-PCR based on TaqMan technology with Takyon No Rox Probe MasterMix dTTP blue 2× (Eurogentec, Liège, Belgium) targeting the open reading frame (ORF) 5 of the *Finistère* strain as previously described [35] and the ADV glycoprotein B gene [34].

qPCR assays were performed on a CFX96 Connect (Bio-Rad). The specificities of the qPCR assays were assessed by analyzing the melting curves of the generated products. Collected samples were normalized internally by simultaneously using the average Cycle Quantification (Cq) of three stable reference genes [36] in each sample (Ribosomal protein S24 (RPS24), ribosomal protein L19 (RPL19) and hypoxanthine phosphoribosyltransferase-1 (HPTR1)) [31,32]. The expression stability of these selected reference genes was assessed as previously described using geNorm [31]. Then, qPCR data (Cq) were subjected to Genex macro analysis (BioRad) [36] and expressed as relative values after Genex macro analysis. Guidelines from [37] for RT-qPCR good practices were followed.

### 2.8. Statistics

**Figure 1:**
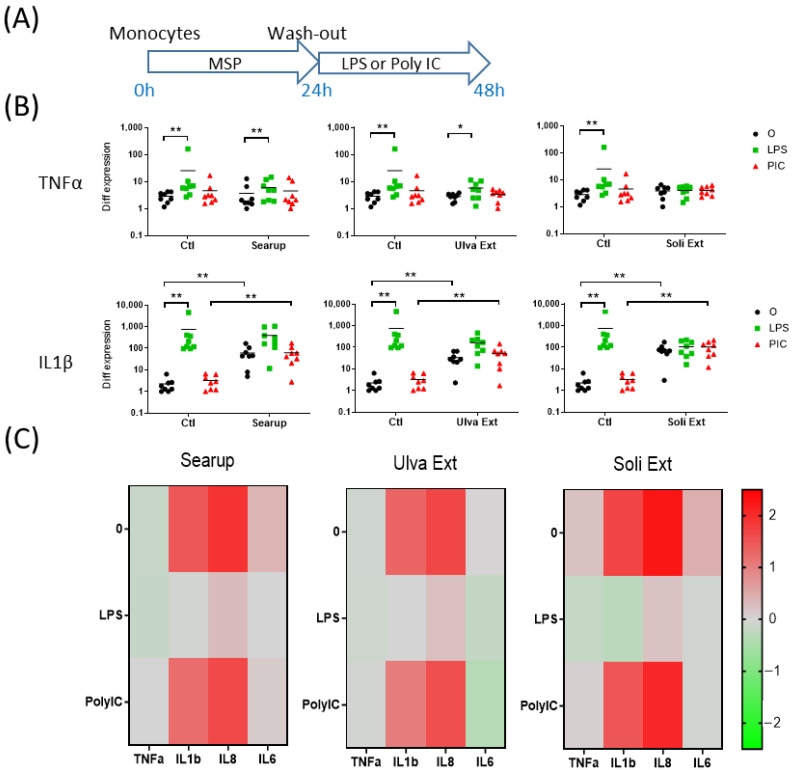
Marine sulfated polysaccharides short-term treated monocytes inflammatory responses to LPS and poly IC. Plastic adherence Monocytes-enriched cells were let untreated (control/Ctl) or treated with the three different MSP^®^ Searup^®^ (Searup^®^), Ulva Extract (Ulva Ext) or Solieria Extract (Soli Ext) for 24 h before washing-out the extract and stimulation with 10 ng/mL LPS or Poly IC for 24 h. TNFα, IL1β, IL8 and IL6 expressions were measured by RT-qPCR. (**A**) Scheme of the experiment, (**B**) TNFα and IL1β transcriptomic expressions, (**C**) Median fold change in untreated or MSP^®^-treated cells, calculated from raw data from B (TNFα, IL1β) and from Supplementary Figure S2B (IL8, IL6). From eight independent experiments and animals. Statistics Wilcoxon paired, non-parametric ranked statistic test, * *p* < 0.05, ** *p* < 0.001.

**Figure 2:**
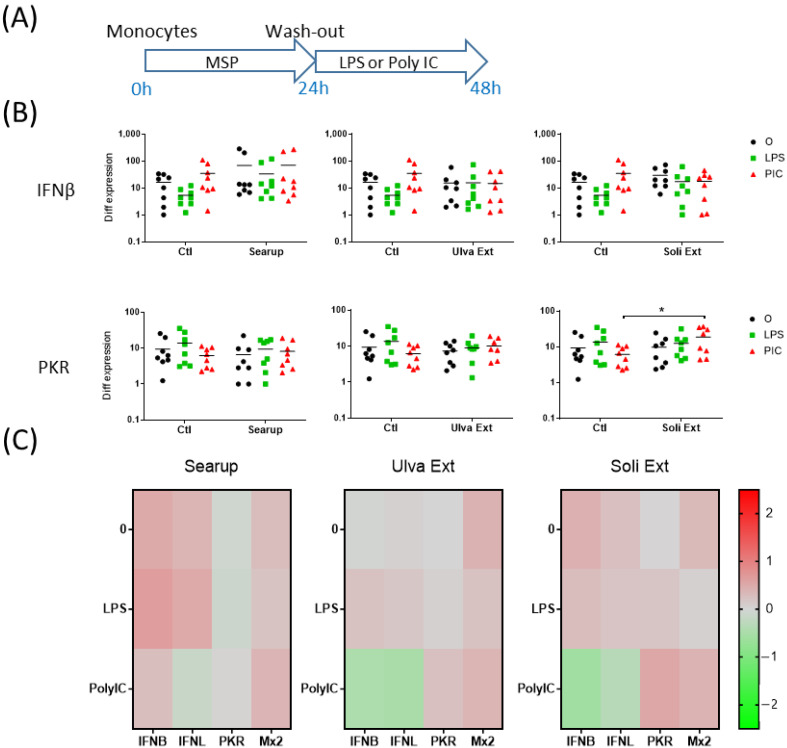
Marine sulfated polysaccharides short-term treated monocytes antiviral responses to LPS and poly IC. Plastic adherence Monocytes-enriched cells were let untreated (control/Ctl) or treated with the three different MSP^®^ Searup^®^ (Searup^®^), Ulva Extract (Ulva Ext) or Solieria Extract (Soli Ext) for 24 h before washing-out the extract and stimulation with 10 ng/mL LPS or Poly IC for 24 h. IFNβ, IFNλ1, PKR and Mx2 expressions were measured by RT-qPCR. (**A**) Scheme of the experiment, (**B**) IFNβ and PKR transcriptomic expressions, (**C**) Median fold change in untreated or MSP^®^-treated cells, calculated from raw data from B (IFNβ, PKR) and from Supplementary Figure S2C (IFNλ1, Mx2). From eight independent experiments and animals. Statistics Wilcoxon paired, non-parametric ranked statistic test, * *p* < 0.05.

**Figure 3:**
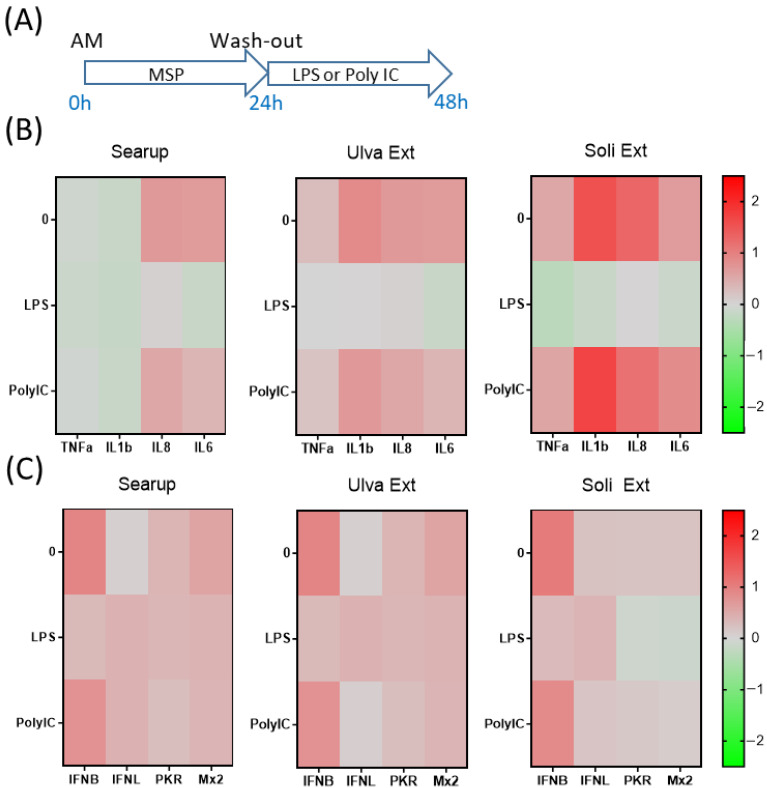
Marine sulfated polysaccharides short-term treated alveolar macrophages responses to LPS and poly IC. Plastic adherence AM-enriched cells were let untreated (control/Ctl) or treated with the three different MSP^®^ Searup^®^ (Searup^®^), Ulva Extract (Ulva Ext) or Solieria Extract (Soli Ext) for 24 h before washing-out the extract and stimulation with 10 ng/mL LPS or Poly IC for 24 h. TNFα, IL1β, IL8, IL6, IFNβ, IFNλ1, PKR and Mx2 expressions were measured by RT-qPCR. (**A**) Scheme of the experiment, (**B**) TNFα, IL1β, IL8 and IL6 median fold change in untreated or MSP^®^-treated cells calculated from raw data from Supplementary Figure S3B, (**C**) IFNβ, IFNλ1, PKR and Mx2 median fold change in untreated or MSP^®^-treated cells, calculated from raw data from Supplementary Figure S3C. From eight independent experiments and animals.

**Figure 4:**
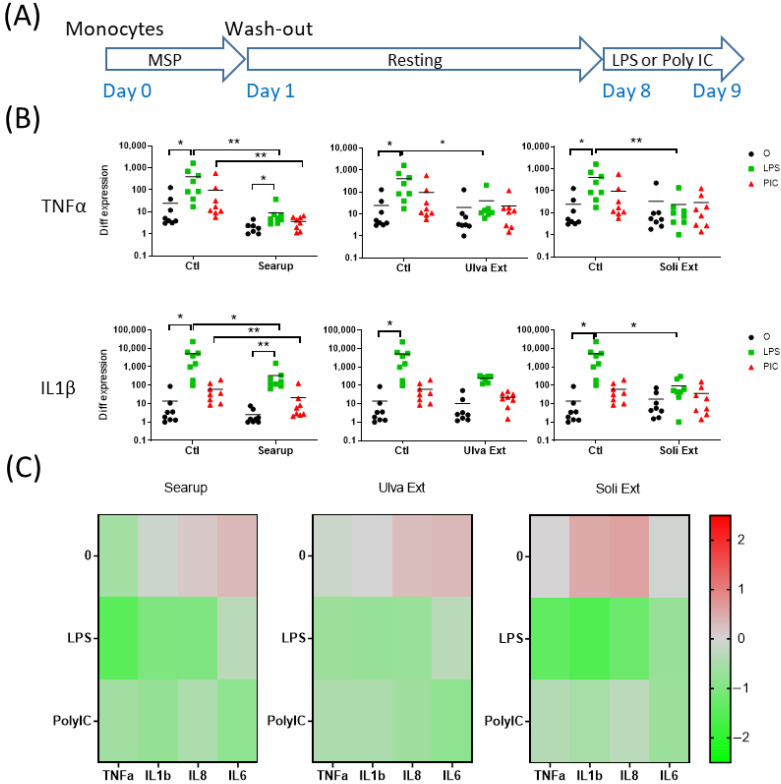
Marine sulfated polysaccharides medium-term treated monocytes inflammatory responses to LPS and poly IC. Plastic adherence Monocytes-enriched cells were let untreated (control/Ctl) or treated with the three different MSP^®^ Searup^®^ (Searup^®^), Ulva Extract (Ulva Ext) or Solieria Extract (Soli Ext) for 24 h before washing-out the extract. Cells rested for 7 days before stimulation with 10 ng/mL LPS or Poly IC for 24 h. TNFα, IL1β, IL8 and IL6 expressions were measured by RT-qPCR. (**A**) Scheme of the experiment, (**B**) TNFα and IL1β transcriptomic expressions, (**C**) Median fold change in untreated or MSP^®^-treated cells, calculated from raw data from B (TNFα, IL1β) and from Supplementary Figure S2E (IL8, IL6). From eight independent experiments and animals. Statistics Wilcoxon paired, non-parametric ranked statistic test, * *p* < 0.05, ** *p* < 0.001.

**Figure 5:**
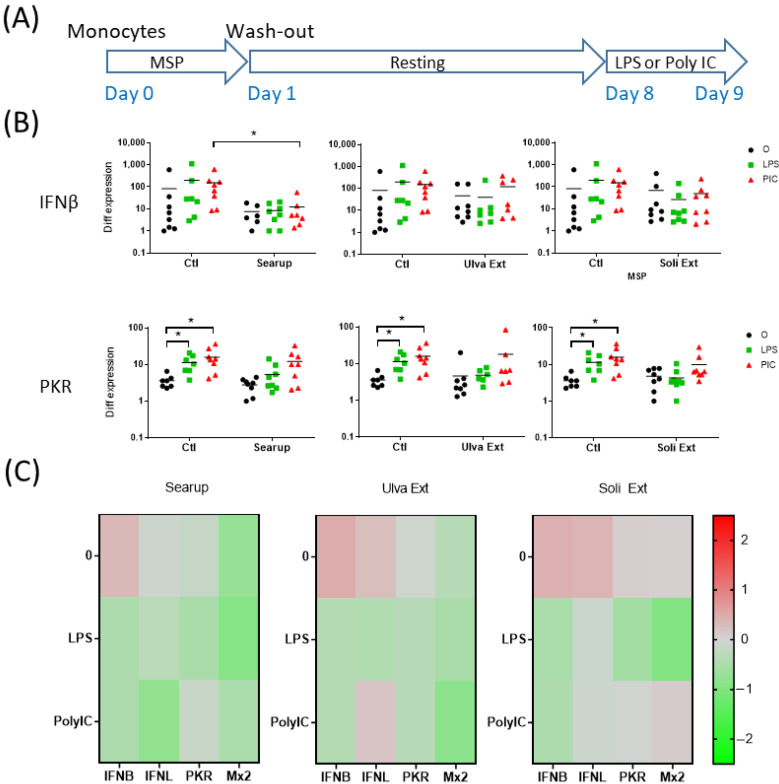
Marine sulfated polysaccharides medium-term treated monocytes antiviral responses to LPS and poly IC. Plastic adherence Monocytes-enriched cells were let untreated (control/Ctl) or treated with the three different MSP^®^ Searup^®^ (Searup^®^), Ulva Extract (Ulva Ext) or Solieria Extract (Soli Ext) for 24 h before washing-out the extract. Cells rested for 7 days before stimulation with 10 ng/mL LPS or Poly IC for 24 h. IFNβ, IFNλ1, PKR and Mx2 expressions were measured by RT-qPCR. (**A**) Scheme of the experiment, (**B**) IFNβ and PKR transcriptomic expressions, (**C**) Median fold change in untreated or MSP^®^-treated cells, calculated from raw data from B (IFNβ, PKR) and from Supplementary Figure S2F (IFNλ1, Mx2). From eight independent experiments and animals. Statistics Wilcoxon paired, non-parametric ranked statistic test, * *p* < 0.05.

**Figure 6:**
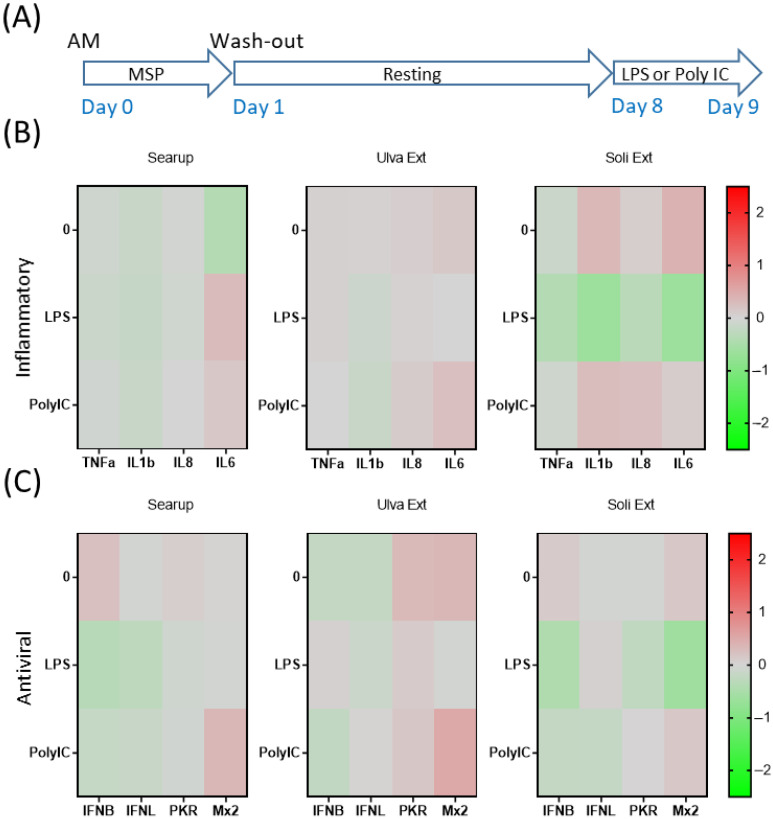
Marine sulfated polysaccharides medium-term treated alveolar macrophages responses to LPS and poly IC. Plastic adherence AM-enriched cells were let untreated (control/Ctl) or treated with the three different MSP^®^ Searup^®^ (Searup^®^), Ulva Extract (Ulva Ext) or Solieria Extract (Soli Ext) for 24 h before washing-out the extract. Cells rested for 7 days before stimulation with 10 ng/mL LPS or Poly IC for 24 h. TNFα, IL1β, IL8, IL6, IFNβ, IFNλ1, PKR and Mx2 expressions were measured by RT-qPCR. (**A**) Scheme of the experiment, (**B**) TNFα, IL1β, IL8 and IL6 median fold change in untreated or MSP^®^-treated cells calculated from raw data from Supplementary Figure S4B, (**C**) IFNβ, IFNλ1, PKR and Mx2 median fold change in untreated or MSP^®^-treated cells, calculated from raw data from Supplementary Figure S4C. From eight independent experiments and animals.

The Wilcoxon paired, non-parametric ranked statistic test was applied using Graphpad Prism 7 (GraphPad Software version 7.0, San Diego, CA, USA), to compare the conditions one to one. Heat Maps depicted the median of the ratios between control or MSP^®^ treated monocytes (Figure 1C, Figure 2C, Figures 4C and 5C) or AM (Figure 3A,B and Figure 6A,B) or the ratios between 0 or LPS treated, or 0 and Poly IC treated monocytes (Supplementary Figure S1A) or AM (Supplementary Figure S1B).

## 3. Results

For monocytes, the complete data of 2 pro-inflammatory cytokines, TNFα and IL1β and 2 antiviral genes, IFNβ and PKR will be depicted in the main figures, together with a heat map depicting the effect of the MSP^®^ (fold changes compared to control, non-MSP^®^ treated monocytes) on the transcriptomic expressions of all of the cytokines tested. For AM, only the heat maps will be depicted in the main figures. The raw data from which were calculated the heat maps are presented in Supplementary Figures S2–S4.

The short-term effects of the different MSP^®^: Ulva extract and Solieria extract, as well as the formulated version of Ulva extract, Searup^®^, were first investigated on porcine peripheral blood monocytes. Monocytes-enriched plastic-adherent cells were cultured for 24 h in presence of MSP^®^. The adherent cells were then washed to remove the MSP^®^ and stimulated for 24 h with 10 ng/mL of LPS, mimicking Gram- bacterial cell wall, or Poly IC, mimicking double-strand virus RNA genome (Figure 1A and Figure 2A). The transcriptomic regulation of 4 pro-inflammatory genes, TNFα, IL1β, IL8 and IL6, were first monitored. Control-monocytes, not treated with MSP^®^, responded to LPS stimulation through upregulation of TNFα, IL1β and IL8 (Figure 1B, Figures S1A and S2B) but did not respond to Poly IC. The 24 h incubation with each of the MSP^®^ products increased IL1β and IL8 basal expression but did not modify the monocyte-response to LPS (Figure 1B,C). Searup^®^, Ulva extract and Solieria extract presented very similar pro-inflammatory properties on peripheral blood monocytes (Figure 1C and Figure 2C). The transcriptomic regulation of four antiviral genes, IFNβ, IFNλ1, PKR and Mx2, were then monitored. In basal conditions, no expression modification was observed for these genes with any of the treatments. Upon LPS treatment, an increase in IFNβ transcripts was observed in Searup^®^ treated monocytes compared with untreated ones, while Poly IC stimulation increased PKR transcripts in Solieria extract treated monocytes compared with non-treated ones (Figure 2B,C).

The short-term action of the three different MSP^®^ products on AM were then investigated. LPS stimulation triggered IL1β, IL8 and IL6 expressions on control-AM (Supplementary Figures S1B and S3B). MSP^®^-treated AM reacted similarly to monocytes with a globally lower impact of MSP^®^ on AM pro-inflammatory-gene regulations (Figure 3B and Figure S3B). Regarding antiviral genes, control AM responded by IFNβ upregulation upon Poly IC stimulation (Supplementary Figures S1B and S3C). The 24-hour-treatments of AM with Ulva extract and Solieria extract allowed steady state IFNβ increase as well as Poly IC mediated induction of PKR (Figure 3C and Figure S3C).

In order to probe the immune training or immune tolerance capacities of MSP^®^, the medium-term effects of the three MSP^®^ products were investigated. Peripheral blood monocytes and AM were MSP^®^-treated for 24 h before washing-out the MSP^®^ and culturing the cells for 7 days. Rested cells were then stimulated 24 h with LPS or Poly IC (Figure 4A, Figure 5A and Figure 6A).

The LPS stimulation of 8-day-cultured control-monocytes triggered TNFα, IL1β, IL8 and IL6 (Figure 4B,C, Figures S1C and S2E). In the same conditions, the Poly IC stimulation triggered upregulations of IL8 and IL6 as well as a tendency to upregulate IL1β (Figure 4B,C, Figures S1C and S2E). The three MSP^®^ products, Searup^®^, Ulva extract and Solieria extract presented very similar capacities to down modulate the LPS and Poly IC-stimulated inflammatory responses of peripheral blood monocytes (Figure 4B,C and Figure S2E). The antiviral response of 7-day-cultured monocytes was then evaluated by monitoring the transcriptomic expressions of INFβ, IFNλ1, PKR and Mx2 genes. Upon LPS and Poly IC stimulations only PKR presented an upregulation (Figure 5B,C and Figure S2F). Again, MSP^®^ treatments triggered some gene down-modulations, Searup^®^ down-modulated the IFNβ and IFNλ1 expressions, whereas Solieria extract presented a tendency to diminish the LPS-induced PKR response (Figure 5B,C and Figure S2F).

The medium-term cultured AM responded strongly to LPS stimulation by TNFα, IL1β, IL8 and IL6 upregulations, whereas Poly IC had no effect on AM pro-inflammatory response (Figure S1D and Figure 6B,C). Of the 3 MSP^®^ products, only Solieria extract presented down-modulatory impact on the LPS-mediated inflammatory response (Figure 6B and Figure S4B). In the medium-term culture of AM, IFNβ and PKR antiviral genes were upregulated upon LPS stimulation (Figure S1D and Figure 6C). Similarly, to the pro-inflammatory response, Solieria extract treatment down-modulated the AM antiviral response (Figure 6C and Figure S4C).

**Figure 7:**
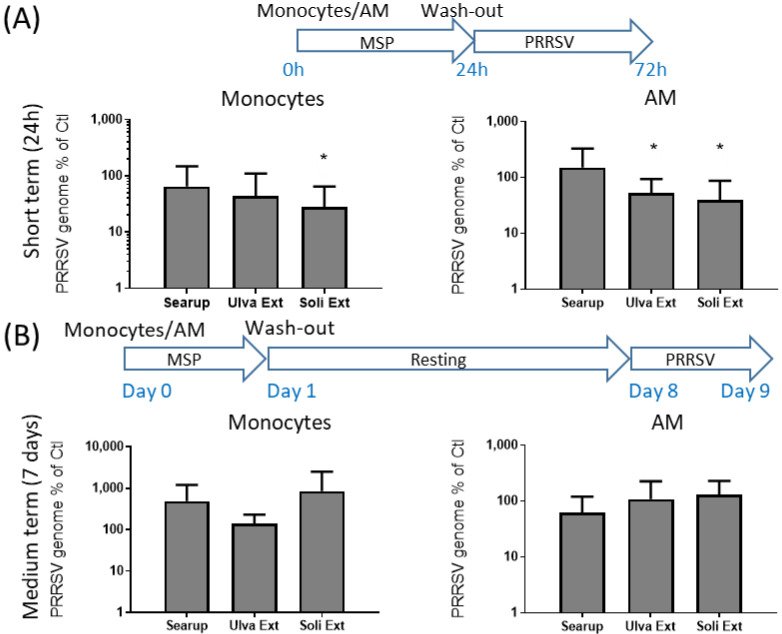
Susceptibility to PRRSV infection of Marine sulfated polysaccharides-treated Monocytes and Alveolar macrophages. Plastic adherence monocytes or AM-enriched cells were let untreated (control/Ctl) or treated with the three different MSP^®^ Searup^®^ (Searup^®^), Ulva Extract (Ulva Ext) or Solieria Extract (Soli Ext) for 24 h before washing-out the extract. (**A**) cells were directly infected with 0.1 (monocytes) or 0.001 (AM) MOI of PRRSV for 48 h before cellular RNA extraction and PRRSV genome dosage using RT-qPCR. (**B**) Cells were rested for 7 days before infection with 0.1 (monocytes) or 0.001 (AM) of PRRSV for 24 h before cellular RNA extraction and PRRSV genome dosage using RT-qPCR. The ratio between MSP^®^ treated and untreated cells are depicted. From eight independent experiments and animals. Statistics Wilcoxon paired, non-parametric ranked statistic test, * *p* < 0.05.

**Figure 8:**
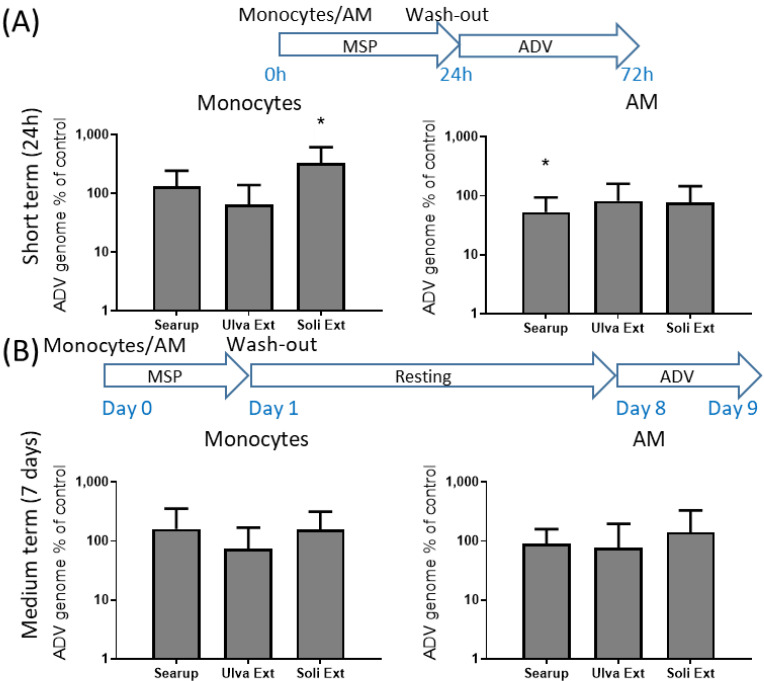
Susceptibility to ADV infection of Marine sulfated polysaccharides-treated Monocytes and Alveolar macrophages. Plastic adherence monocytes or AM-enriched cells were let untreated (control/Ctl) or treated with the three different MSP^®^ Searup^®^ (Searup^®^), Ulva Extract (Ulva Ext) or Solieria Extract (Soli Ext) for 24 h before washing-out the extract. (**A**) cells were directly infected with 0.1 (monocytes) or 0.001 (AM) MOI of PRRSV for 48 h before cellular RNA extraction and PRRSV genome dosage using RT-qPCR. (**B**) Cells were rested for 7 days before infection with 0.1 (monocytes) or 0.01 (AM) of ADV for 24 h before cellular RNA extraction and ADV genome dosage using RT-qPCR. The ratio between MSP^®^ treated and untreated cells are depicted. From eight independent experiments and animals. Statistics Wilcoxon paired, non-parametric ranked statistic test, * *p* < 0.05.

The observed modifications of the response to immune stimuli upon treatment with different MSP^®^ products raised the question of the impact on the viral infection of monocytes and macrophages. Two viruses infecting monocytes and macrophages were tested, PRRSV and ADV. Short-term cultured monocytes and AM were infected, respectively, at MOI 0.1 and MOI 0.001 for 48 h. As expected, monocytes susceptibility to PRRSV replication was very low (Cq = 28.7 +/− 3.7) compared with that of AM (Cq = 17.2 +/− 3.0), despite the 2 logs differences in the infective dose. Monocytes treatment with Ulva extract presented a tendency to decrease PRRSV replication down to 43% of the replication in control monocytes. Treatment with Solieria extract decreased the PRRSV replication in monocytes down to 28% of the control cells (Figure 7A). AM treatment with both Ulva extract and Solieria extract decreased the PRRSV replication in AM to 56% and 40%, respectively (Figure 7A). ADV-infected short-term cultured monocytes and AM presented similar Cq outcomes, respectively, Cq = 26.9 +/− 4.1 and Cq = 24.0 +/− 3.1, despite the 1 log difference in the infective dose (respectively, MOI 0.1 and 0.01). Treatment of monocytes with Searup^®^ and Ulva extract did not significantly modify the cell susceptibility to ADV infection; however, treatment with Solieria extract increased the susceptibility to ADV to 326% compared to the control cells (Figure 8A). Treatment of AM with Searup^®^ decreased susceptibility to 53% of the control cells however treatment with Ulva extract and Solieria extract did not significantly modify the susceptibility of the cells to ADV infection, (Figure 8A).

Medium-term cultured monocytes and AM were infected at MOI 0.1 for 24 h, instead of 48 h for the short-term cultured cells. Medium-term cultured monocytes were even less susceptible to PRRSV infection than their AM counterpart (respectively, Cq = 30.7 +/− 4.1 and Cq = 19.9 +/− 4.8). No significant effect of MSP^®^ on PPRSV infection was observed at this time point neither for monocytes nor for AM (Figure 7B). Medium-term cultured monocytes were far less susceptible to ADV than their AM counterpart (respectively, Cq = 30.5 +/ − 4.2 and 24.7 +/− 3.5) according to the 1 log differences in the infective dose (respectively, MOI 0.1 and 0.01). No significant effect of MSP^®^ on ADV infection was observed at this point for monocytes as well as for AM (Figure 8B).

## 4. Discussion

Respiratory infections are a major cause of porcine morbidity and mortality worldwide [1]. PRRSV is currently one of the most important respiratory viruses in pigs [38]. Available vaccines are mainly effective on autologous infections, mostly by reducing the clinical signs without blocking the viral transmission. Thus, this virus is still having a major impact on pig wellbeing and productivity in most countries [38]. Moreover, PRRSV infections enables bacterial superinfections requiring the use of antimicrobials to control bacteria [1]. As a result of the development and the spread of antimicrobial resistance there is a strong need for alternatives to common antimicrobial treatments. Algae contain a high diversity of biologically active molecules with novel chemical structures and interesting pharmacological activities [8]. The data presented in this study revealed that the different MSP^®^ tested gave a similar steady state increase in the transcriptomic early expression of IL1β and IL8 (typical anti-inflammatory cytokine and chemokine) on monocytes without affecting the antiviral response. Conversely, the impact of the MSP^®^ on AM was greater with Solieria extract than with Ulva extract and Searup^®^, and this was the case for a larger set of cytokines: TNFα, IL1β, IL8, IL6 and IFNβ. Despite these differences, the impact of the MSP^®^ treatments on the different cell types (monocytes versus AM) was overall similar between the MSP^®^. We previously observed a similar pro-inflammatory effect of Ulva extract on porcine intestinal epithelial cells [18]. Although the MSP^®^ components responsible of this effect remain to be identified. This pro-inflammatory impact of MSP^®^, through IL1β and IL8 cytokines upregulation is clearly observed at the basal level but not after LPS or Poly IC stimulation. We can speculate that MSP^®^ exposed cells reach their maximal level of stimulation, rending LPS or Poly IC sur-stimulations ineffective or that MSP^®^ exposition would exhaust the pro-inflammatory signaling. Interestingly, only the short-term treatment of monocytes with Solieria extract increased the Poly IC-mediated PKR response with a parallel increase in the monocytes resistance to PRRSV infection. For short-term AM treatments both Ulva extract and Solieria extract increased the Poly IC-mediated PKR response and presented a clear action against the PRRSV infection. Poly IC mimics the double stranded RNA synthesized during the PRRSV infection cycle [39], and the observed increased Poly IC antiviral response upon Ulva extract and Solieria extract treatments might mimic what occurs upon PRRSV infection. The stronger antiviral impact on AM can be attributed to the higher susceptibility of AM to PRRSV infection, that might allow the MSP^®^ inhibitory effect to be more visible. Interestingly, for ADV, a DNA virus, Solieria extract treatment facilitated ADV replication in monocytes, whereas some antiviral effect is observed for Searup^®^ treatment of AM. This is not the first time that antiviral effects have been demonstrated for algal extracts. Indeed, previous reports show direct and indirect antiviral effects of algal extracts, mostly due to sulfated polysaccharides, such as ulvan for green algae or carrageenan, galactan and agar for red algae (for review see [40]), acting against several viruses such as human metapneumovirus (HMPV), Newcastle disease virus (NDV), Marek’s disease virus (MDV) and human immunodeficiency virus (HIV) [41,42,43,44].

When looking at longer time point post MSP^®^ treatments, MSP^®^ impact appeared opposed to their short-term effect, with a downmodulation of the LPS and Poly IC responses and a more marked effect on monocytes than on AM. According to Divangahi et al. [23], this is a typical induction of immune tolerance, the opposite of immune training. Interestingly this tolerance has no effect on the replication of the two viruses tested, PRRSV and ADV. Since the detrimental effect on animal growth by persistent viral infections such as PRRSV disease are mainly mediated through the chronical inflammatory state of the animals, the short-term anti-PRRSV action of Ulva extract associated with its median term anti-inflammatory action might bring a double benefit in chronically PRRSV-infected farms.

Among the MSP^®^ tested here, Searup^®^ is the only one already commercialized. One recommended application is 1 day before and 2 days after vaccination protocols as a co-adjuvant that could consolidate the vaccine-induced immune reaction. The transient pro-inflammatory impact of Searup^®^ observed here is in agreement with this prescription. Another recommended application of Searup^®^ is a 5-day-treatment in case of high sanitary pressure. Again, the transient pro-inflammatory impact of Searup^®^ might facilitate the development of the immune response as in the case of vaccination, but the medium-term down-modulatory effects we observed might also present a beneficial impact by resolving the late infection-mediated inflammatory clinical signs, associated with a more rapid recovery. It would be of great interest to confirm these results in the field.

## 5. Conclusions

In the current study, we studied the short- and median-term impact of three algal extracts, in vitro, on the pro-inflammatory and antiviral responses of porcine primary blood monocytes and alveolar macrophages. Then, we tested the susceptibility of the treated cells to infection by PRRSV and ADV. All extracts presented a pro-inflammatory short-term effect, associated with two of them, with an inhibition of the PRRSV replication. Inversely, the three extracts presented an anti-inflammatory median term effect without altering PRRSV replication. These observations prompted us to conclude that the in vivo testing of the anti-PRRSV properties of the algal extracts is warranted.

## Figures and Tables

**Table 1:** Seaweed extract compositions.

Extracts	DM%	MM%	OM%	pH	C (%DM)	N (%DM)	S (%DM)
Ulva	13.4%	50.1%	49.9%	2.6	18.5%	1.6%	9.8%
Soleria	16.1%	62.7%	37.3%	2.6	14.4%	2.9%	5.3%

DM: Dry Matter; MM: Mineral Matter; OM: Organic Matter.

**Table 2:** RT-qPCR primers.

Genes	Sequences (5′-3′)	Size (bp)	Effic.	Tm (°C)	References
***Cytokines***					
TNFα	F: TGGTGGTGCCGACAGATG R: CAGCCTTGGCCCCTGAA	64	0.96	64	[29]
IL1β	F: TGCCAACGTGCAGTCTATGG R: TGGGCCAGCCAGCACTAG	70	0.89	60	NM_214055
IL8/CXCL8	F: CCGTGTCAACATGACTTCCAA R: GAGCTGCAGAAAGCAGGAAAA	65	0.97	60	NM_213867
IL6	F: CTGCTTCTGGTGATGGCTACTG R: GGCATCACCTTTGGCATCTT	69	0.94	60	[29]
IFNβ	F: TGTGGAACTTGATGGGCAGA R: GAATGGTCATGTCTCCCCTGGT	92	0.98	60	MH538100
IFNλ1	F: GAGGCTGAGCTAGACTTGAC R: CCTGAAGTTCGACGTGGATG	115	0.95	60	[30]
PKR	F: CACATCGGCTTCAGAGTCAG R: GGGCGAGGTAAATGTAGGTG	166	0.96	61	[31]
Mx2	F: CCGACTTCAGTTCAGGATGG R: ACAGGAGACGGTCCGTTTAC	156	1.03	62	[31]
***References***					
RPS24	F: AAGGAACGCAAGAACAGAATGAA R: TTTGCCAGCACCAACGTTG	62	0.95	60	[32]
RPL19	F: AACTCCCGTCAGCAGATCC R: AGTACCCTTCCGCTTACCG	147	0.90	60	[30]
HPRT1	F: CTGCACCTCCGCCTCTC R: TCACTAATCACGACGCTGGG	119	0.90	60	XM_021079504
***Viruses***					
PRRSV	F: TATGCGAGCTGAATGGGACC R: AGGATATGAGTGGCAACCGG P: HEX-TGGGCAGTTGAGACTTTCGTGCT-TAMRA	/		60	[33]
ADV	F: GCGGGTACGTGTACTACGAG R: GAGGCCCTGGAAGAAGTTGG P: 6FAM-ACTACAGCTACGTGCGCATGGTGGAG-TAMRA	287		63	[34]

## Data Availability

Data is contained within the article or supplementary material.

## Supplementary Materials

The following supporting information can be downloaded at: https://www.mdpi.com/article/10.3390/ani12192576/s1, Figure S1: Heat map depicting the ratios between 0 or LPS treated, or 0 and Poly IC treated monocytes or AM; Figure S2: Raw data (monocytes) from which were calculated the different heat maps; Figure S3: Raw data (alveolar macrophages) from which were calculated the different heat maps. Figure S4: Raw data (alveolar macrophages 2) from which were calculated the different heat maps.
