# Supplementary material for: Marine-Sulfated Polysaccharides Extracts Exhibit Contrasted Time-Dependent Immunomodulatory and Antiviral Properties on Porcine Monocytes and Alveolar Macrophages
Source: Animals (Basel). 2022 Sep 27;12(19):2576. doi: 10.3390/ani12192576 (PMC9559208; doi:10.3390/ani12192576)
Supplement: Supplementary file 1 [file animals-12-02576-s001.zip › Hervet_Olmix_SuppFigS1_revised.pptx]

## Slide 1
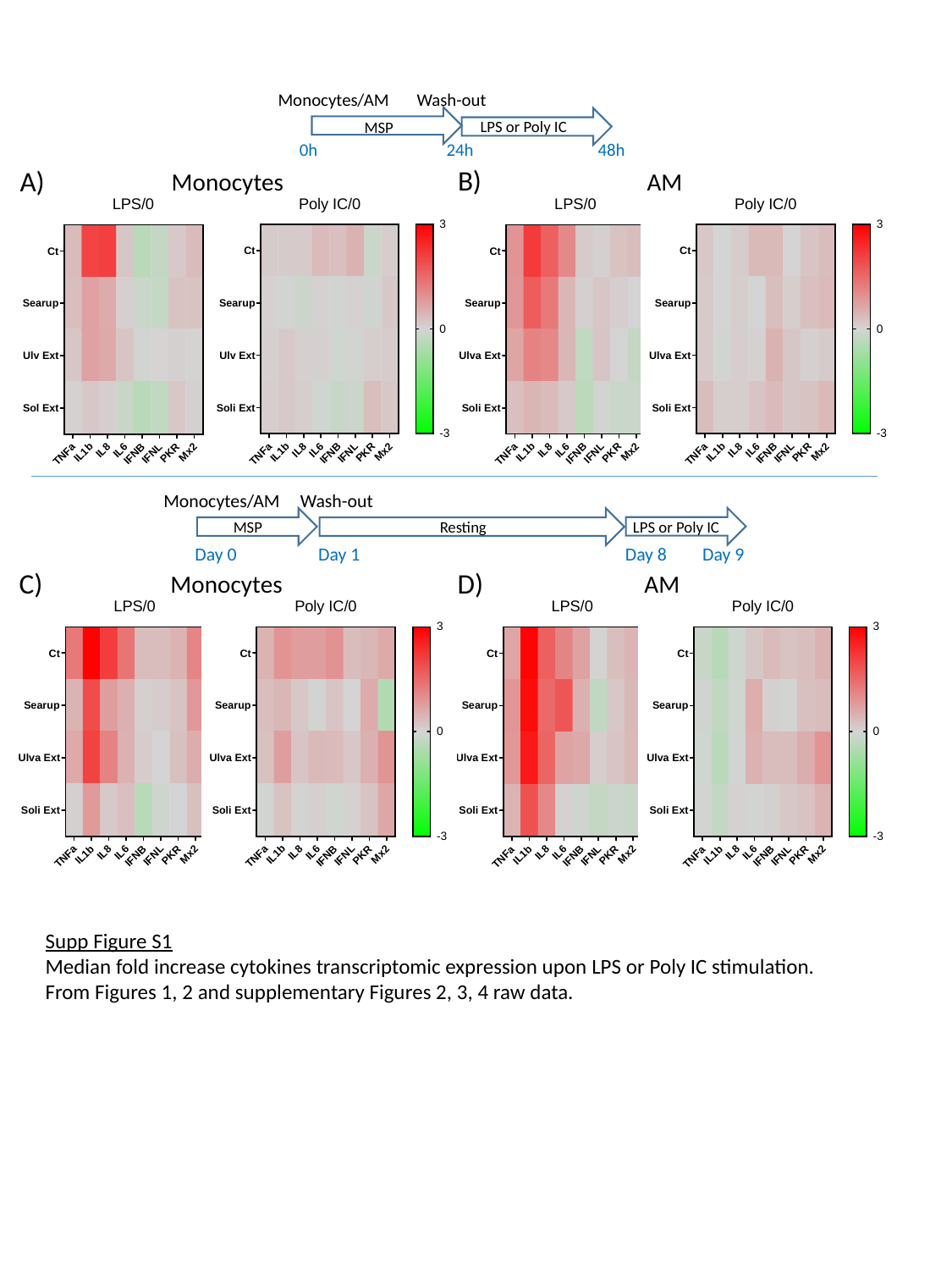

Wash-out
Monocytes/AM
LPS or Poly IC
MSP
48h
24h
0h
B)
A)
AM
Monocytes
Wash-out
Monocytes/AM
MSP
Resting
LPS or Poly IC
Day 8
Day 0
Day 9
Day 1
C)
D)
Monocytes
AM
Supp Figure S1
Median fold increase cytokines transcriptomic expression upon LPS or Poly IC stimulation.
From Figures 1, 2 and supplementary Figures 2, 3, 4 raw data.
