# Supplementary material for: Marine-Sulfated Polysaccharides Extracts Exhibit Contrasted Time-Dependent Immunomodulatory and Antiviral Properties on Porcine Monocytes and Alveolar Macrophages
Source: Animals (Basel). 2022 Sep 27;12(19):2576. doi: 10.3390/ani12192576 (PMC9559208; doi:10.3390/ani12192576)
Supplement: Supplementary file 1 [file animals-12-02576-s001.zip › Hervet_Olmix_SuppFigS2_revised.pptx]

## Slide 1
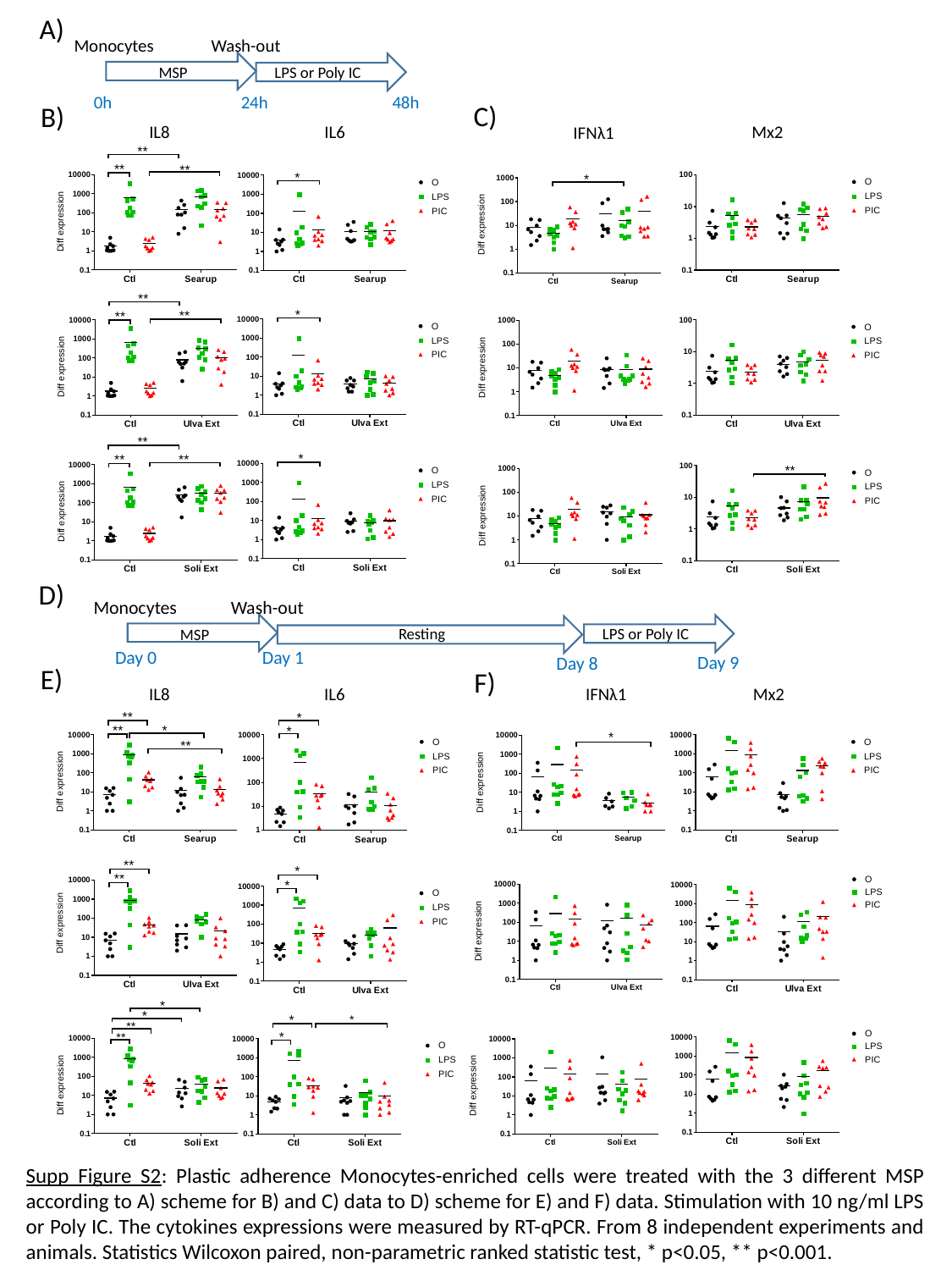

A)
Wash-out
Monocytes
LPS or Poly IC
MSP
48h
24h
0h
C)
B)
Mx2
IL8
IL6
IFNλ1
D)
Wash-out
Monocytes
Resting
LPS or Poly IC
MSP
Day 1
Day 0
Day 9
Day 8
E)
F)
IFNλ1
IL8
IL6
Mx2
Supp Figure S2: Plastic adherence Monocytes-enriched cells were treated with the 3 different MSP according to A) scheme for B) and C) data to D) scheme for E) and F) data. Stimulation with 10 ng/ml LPS or Poly IC. The cytokines expressions were measured by RT-qPCR. From 8 independent experiments and animals. Statistics Wilcoxon paired, non-parametric ranked statistic test, * p<0.05, ** p<0.001.
